# Supplementary material for: Peptides for Coating TiO2 Implants: An In Silico Approach
Source: Int J Mol Sci. 2022 Nov 14;23(22):14048. doi: 10.3390/ijms232214048 (PMC9693858; doi:10.3390/ijms232214048)
Supplement: Supplementary file 1 [file ijms-23-14048-s001.zip › S1.pdf]

Table S1. Docking analysis of the integrins involved in the osseointegration process with the proteins containing RGD motif. In this matter, docking analysis showed that fibrinogen is the biological ligand with the greater affinity binding with α5β1, αvβ3 and αIIbβ3 integrins (-324.1, -334.7 and -271.3, respectively). It is important to note that the VdW+Elec scoring unable to distinguish between favorable and poor protein-ligand interactions. The score represents the potential energy change of the protein and ligand bind. In other words, a very negative score corresponds to a strong binding while a less negative corresponds to a weak binding.

Integrins (Receptors)	Proteins containing RGD (Ligands)	(VdW+Elec)
	Fibronectin	-281.3
α5β1	Vitronectin	-227.3
	Fibrinogen	-324.1
	Fibronectin	-280.9
αvβ3	Vitronectin	-230.5
	Fibrinogen	-334.7
	Fibronectin	-231.0
αIIbβ3	Vitronectin	-197.1
	Fibrinogen	-271.3

Table S2. Docking analysis of the integrins involved in the osseointegration process with low-cost proteins. Myosin was the protein that presented the best binding results with integrins α5β1, αvβ3 and αIIbβ3 (-395.1, -397.8 and -429.1, respectively).

Integrins (Receptors)	Proteins containing RGD (Ligands)	(VdW+Elec)
	Bovine Serum Albumin	-315.5
α5β1	Ovine Serum Albumin	-319.6
	Myosin	-395.1
	Bovine Serum Albumin	-351.8
αvβ3	Ovine Serum Albumin	-336.7
	Myosin	-397.8
	Bovine Serum Albumin	-243.0
αIIbβ3	Ovine Serum Albumin	-256.8
	Myosin	-429.1

Table S3. Modelled motifs and peptides.

**Peptide**	**ID**	**3DRefiner (score)**	**Ramachandran**
RGD	V04754526462078	181.172	100.00%
LKA	C21705436136961	251.872	100.00%
PDG	W01960734338045	198.619	99.00%
DGL	X24210131361008	145.249	100.00%
TGR	X24773544619083	252.258	100.00%

QPYLFATDSLIKRGD	R05411144699097	1045.07	92.31%
QPYLFATDSLIKLKA	V05783641668081	1199.97	92.31%
QPYLFATDSLIKPDG	L06094350385904	1097.43	92.31%
QPYLFATDSLIKDGL	W21962160466909	1224.39	84.62%
QPYLFATDSLIKTGR	T06467014542103	1075.45	92.31%
GHTHYHAVRTQTRGD	A06868278309107	1116.52	92.31%
GHTHYHAVRTQTLKA	J07287096941948	1026.62	92.31%
GHTHYHAVRTQTPDG	V07649126194000	1373.60	92.31%
GHTHYHAVRTQTDGL	Q08008346199989	1229.63	61.54%
GHTHYHAVRTQTTGR	K13098217226982	1040.59	84.62%
RKLPDARGD	H13344499234915	589.390	85.71%
RKLPDALKA	X13528307888985	580.441	57.14%
RKLPDAPDG	N14177775423050	526.348	85.71%
RKLPDADGL	X14555980346918	767.398	85.71%
RKLPDATGR	D14803003139019	552.638	85.71%
GVL	F25401392591953	230.285	100.00%
VLP	I26060282946110	218.742	100.00%
KDD	X26801342442989	102.173	100.00%
DRG	E27266373552084	157.916	100.00%
QPYLFATDSLIKGVL	P15152441345930	1072.04	92.31%
QPYLFATDSLIKVLP	N15953168220997	1061.95	76.92%
QPYLFATDSLIKKDD	E16599323319912	1068.90	100.00%
QPYLFATDSLIKDRG	C17391386338949	1126.06	84.62%
GHTHYHAVRTQTGVL	U17790981741905	1140.65	84.62
GHTHYHAVRTQTVLP	F18383252851009	1281.99	69.23%
GHTHYHAVRTQTKDD	I20667215733051	1309.71	69.23%
GHTHYHAVRTQTDRG	T29047415877104	1371.88	76.92%
RKLPDAGVL	W29681113162994	629.480	71.43%
RKLPDAVLP	C23791329088926	556.624	85.71%
RKLPDAKDD	K30104607878923	567.133	57.14%
RKLPDADRG	X30655410418034	690.092	85.71%
DSQ	X28246158435106	160.017	100.00%
QAG	V28650720541000	160.360	100.00%
MDS	G29125438069105	427.278	100.00%
DLN	R29574049352884	202.319	100.00%
QPYLFATDSLIKDSQ	E31178552998066	1041.97	84.62%
QPYLFATDSLIKQAG	K31780036655903	1104.53	92.31%
QPYLFATDSLIKMDS	A32184160361052	1042.77	100.00%
QPYLFATDSLIKDLN	W32545390818119	1037.89	76.92%
GHTHYHAVRTQTDSQ	I00812461910009	937.670	84.62%
GHTHYHAVRTQTQAG	A01141656599998	1268.42	76.92%
GHTHYHAVRTQTMDS	R01599465622902	1256.76	69.23%
GHTHYHAVRTQTDLN	O25123734586954	1100.70	83.33%
RKLPDADSQ	Q02044124416113	512.814	85.71%
RKLPDAQAG	H02519593763113	615.015	100.00%
RKLPDAMDS	R03405769977093	725.995	85.71%
RKLPDADLN	H04046648834944	501.472	85.71%

Table S4. The molecular docking results of the 53 modelled peptides with each integrin. The ten peptides that demonstrated best affinity with the integrins are highlighted.

Peptide	Integrin		
	**α5β1**	**αvβ3**	**αIIbβ3**
RGD	-155,5	-163,5	-150,4
LKA	-168,6	-155,9	-165,3
PDG	-104,5	-105	-101,9
DGL	-91,2	-92,1	-92,7
TGR	-169,7	-158,5	-152,5
QPYLFATDSLIKRGD	-147,9	-168,9	-147
**QPYLFATDSLIKLKA**	**-215,4**	**-224**	**-223,3**
QPYLFATDSLIKPDG	-173,9	-145,6	-136,5
QPYLFATDSLIKDGL	-131,2	-145,6	-149,4
QPYLFATDSLIKTGR	-182	-178,1	-186,4
**GHTHYHAVRTQTRGD**	**-204,2**	-185,5	**-213,5**
**GHTHYHAVRTQTLKA**	**-198,1**	-181,9	**-213,7**
GHTHYHAVRTQTPDG	-189,2	**-208,8**	-179,8
GHTHYHAVRTQTDGL	-166,2	-163,9	-159
**GHTHYHAVRTQTTGR**	**-238,2**	**-219**	**-242,6**
**RKLPDARGD**	**-217,4**	**-216,3**	**-196,6**
**RKLPDALKA**	**-227**	**-234,4**	**-221,9**
RKLPDAPDG	-130,7	-142,3	-127,6
RKLPDADGL	-133	-149,1	-153,6
**RKLPDATGR**	**-218,1**	**-225,9**	**-235,6**
GVL	-79,1	-81,2	-75,6
VLP	-70,2	-71,3	-66,3
KDD	-118	-123,2	-119,3
DRG	-117,6	-118,6	-119,3
QPYLFATDSLIKGVL	-143,5	-147,6	-139,6
QPYLFATDSLIKVLP	-128,1	-148,6	-141,8
QPYLFATDSLIKKDD	-162	**-207,6**	-172,5
QPYLFATDSLIKDRG	-144,4	-169,1	-142,1
GHTHYHAVRTQTGVL	-185,7	**-210,8**	**-182,6**
GHTHYHAVRTQTVLP	-174,9	-185,8	-181,9
GHTHYHAVRTQTKDD	-145,6	-156,4	-147,4
GHTHYHAVRTQTDRG	-185,5	-194,7	-191,2
RKLPDAGVL	-185,1	-167,8	-176,8
**RKLPDAVLP**	**-195,9**	**-205,3**	**-183,1**
RKLPDAKDD	-127,8	-144,2	-151,9
RKLPDADRG	-191,3	-201,9	-174
DSQ	-109,8	-117,4	-107,3
QAG	-80	-87,9	-78,1
MDS	-107,2	-104,2	-97,5
DLN	-102,5	-112,5	-97,6
QPYLFATDSLIKDSQ	-129,3	-168,2	-130,5
QPYLFATDSLIKQAG	-136,1	-140,4	-124,4
QPYLFATDSLIKMDS	-153,2	-164,9	-146,1
QPYLFATDSLIKDLN	-123,8	-138	-137,1

GHTHYHAVRTQTDSQ	-155,2	-158,5	-141,6
**GHTHYHAVRTQTQAG**	**-204,9**	-194,6	**-200**
GHTHYHAVRTQTMDS	-163,9	-164,5	-150
**GHTHYHAVRTQTDLN**	**-196,4**	**-213,4**	**-202,2**
RKLPDADSQ	-169,9	-195,5	-153,4
RKLPDAQAG	-177,9	-167,8	-158,1
RKLPDAMDS	-171,9	-191,9	-151,9
RKLPDADLN	-176,9	-195	-174,6

Table S5. Assembly of the complex peptides.

Peptide that binds TiO2	Motif recognized by integrin α5β1 (Ligand)	Complete amino acid sequence (complex peptide)	Motif recognized by integrin αvβ3 (Ligand)	Complete amino acid sequence (complex peptide)	Motif recognized by integrin αIIbβ3 (Ligand)	Complete amino acid sequence (complex peptide)
QPYLFATDSLIK	RGD (Fibronectin)	QPYLFATDSLIKRGD	RGD (Fibronectin)	QPYLFATDSLIKRGD	RGD (Fibronectin)	QPYLFATDSLIKRGD
	RGD (Vitronectin)	QPYLFATDSLIKRGD	RGD (Vitronectin)	QPYLFATDSLIKRGD	RGD (Vitronectin)	QPYLFATDSLIKRGD
	RGD (Fibrinogen)	QPYLFATDSLIKRGD	RGD (Fibrinogen)	QPYLFATDSLIKRGD	RGD (Fibrinogen)	QPYLFATDSLIKRGD
	LKA (Bovine Serum Albumin)	QPYLFATDSLIKLKA	GVL (Bovine Serum Albumin)	QPYLFATDSLIKGVL	DSQ (Bovine Serum Albumin)	QPYLFATDSLIKDSQ
	PDG (Ovine Serum Albumin)	QPYLFATDSLIKPDG	VLP (Ovine Serum Albumin)	QPYLFATDSLIKVLP	QAG (Ovine Serum Albumin)	QPYLFATDSLIKQAG
	TGR (Myosin)	QPYLFATDSLIKTGR	DRG (Myosin)	QPYLFATDSLIKDRG	DLN (Myosin)	QPYLFATDSLIKDLN
GHTHYHAVRTQT	RGD (Fibronectin)	GHTHYHAVRTQTRGD	RGD (Fibronectin)	GHTHYHAVRTQTRGD	RGD (Fibronectin)	GHTHYHAVRTQTRGD

	RGD (Vitronectin)	GHTHYHAVRTQTRGD	RGD (Vitronectin)	GHTHYHAVRTQTRGD	RGD (Vitronectin)	GHTHYHAVRTQTRGD
	RGD (Fibrinogen)	GHTHYHAVRTQTRGD	RGD (Fibrinogen)	GHTHYHAVRTQTRGD	RGD (Fibrinogen)	GHTHYHAVRTQTRGD
	LKA (Bovine Serum Albumin)	GHTHYHAVRTQTLKA	GVL (Bovine Serum Albumin)	GHTHYHAVRTQTGVL	DSQ (Bovine Serum Albumin)	GHTHYHAVRTQTDSQ
	PDG (Ovine Serum Albumin)	GHTHYHAVRTQTPDG	VLP (Ovine Serum Albumin)	GHTHYHAVRTQTVLP	QAG (Ovine Serum Albumin)	GHTHYHAVRTQTQAG
	TGR (Myosin)	GHTHYHAVRTQTTGR	DRG (Myosin)	GHTHYHAVRTQTDRG	DLN (Myosin)	GHTHYHAVRTQTDLN
RKLPDA	RGD (Fibronectin)	RKLPDARGD	RGD (Fibronectin)	RKLPDARGD	RGD (Fibronectin)	RKLPDARGD
	RGD (Vitronectin)	RKLPDARGD	RGD (Vitronectin)	RKLPDARGD	RGD (Vitronectin)	RKLPDARGD
	RGD (Fibrinogen)	RKLPDARGD	RGD (Fibrinogen)	RKLPDARGD	RGD (Fibrinogen)	RKLPDARGD
	LKA (Bovine Serum Albumin)	RKLPDALKA	GVL (Bovine Serum Albumin)	RKLPDAGVL	DSQ (Bovine Serum Albumin)	RKLPDADSQ

PDG (Ovine Serum Albumin)	RKLPDAPDG	VLP (Ovine Serum Albumin)	RKLPDAVLP	QAG (Ovine Serum Albumin)	RKLPDAQAG
TGR (Myosin)	RKLPDATGR	DRG (Myosin)	RKLPDADRG	DLN (Myosin)	RKLPDADLN

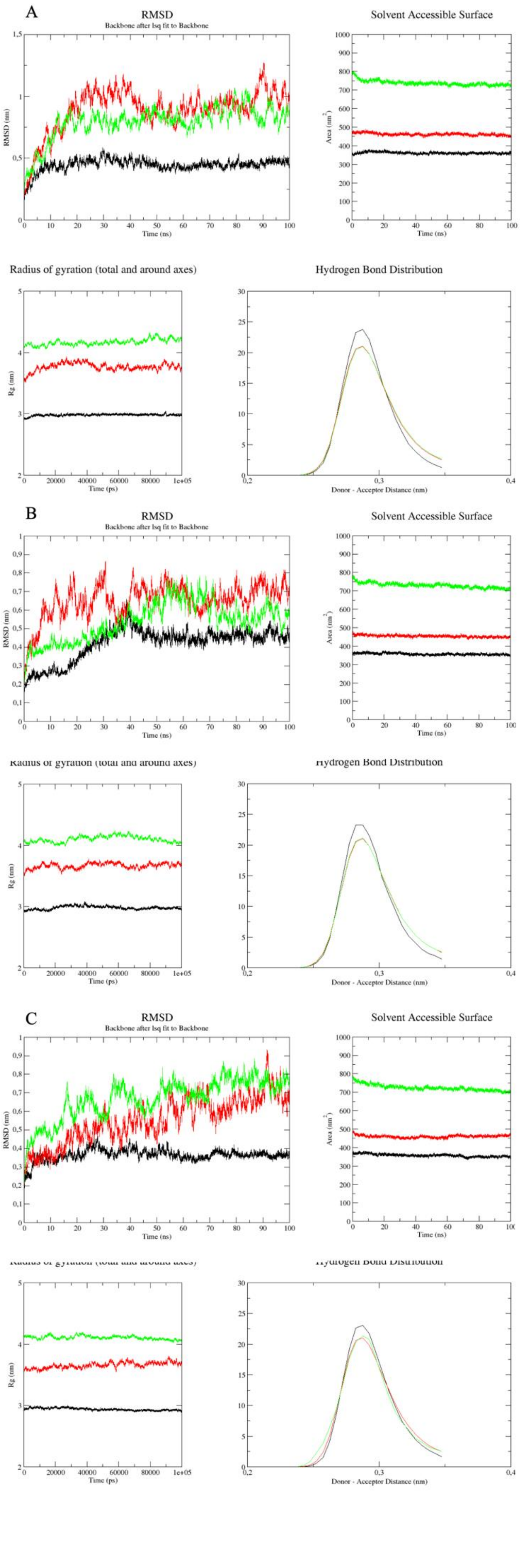
A
RMSD
Backbone after lsq fit to Backbone
RMSD (nm)
Time (ns)
Solvent Accessible Surface
Area (nm2)
Time (ns)
Radius of gyration (total and around axes)
Rg (nm)
Time (ps)
Hydrogen Bond Distribution
Donor - Acceptor Distance (nm)
B
RMSD
Backbone after lsq fit to Backbone
RMSD (nm)
Time (ns)
Solvent Accessible Surface
Area (nm2)
Time (ns)
Rg (nm)
Time (ps)
Donor - Acceptor Distance (nm)
C
RMSD
Backbone after lsq fit to Backbone
RMSD (nm)
Time (ns)
Solvent Accessible Surface
Area (nm2)
Time (ns)
Rg (nm)
Time (ps)
Donor - Acceptor Distance (nm)

D

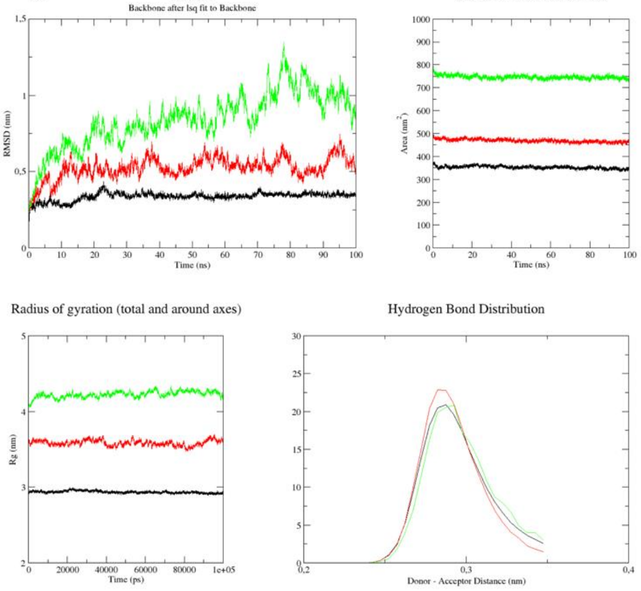
RMSD
Backbone after lsq fit to Backbone
RMSD (nm)
Time (ns)
Solvent Accessible Surface
Area (nm^2)
Time (ns)
Radius of gyration (total and around axes)
Rg (nm)
Time (ps)
Hydrogen Bond Distribution
Donor - Acceptor Distance (nm)

E

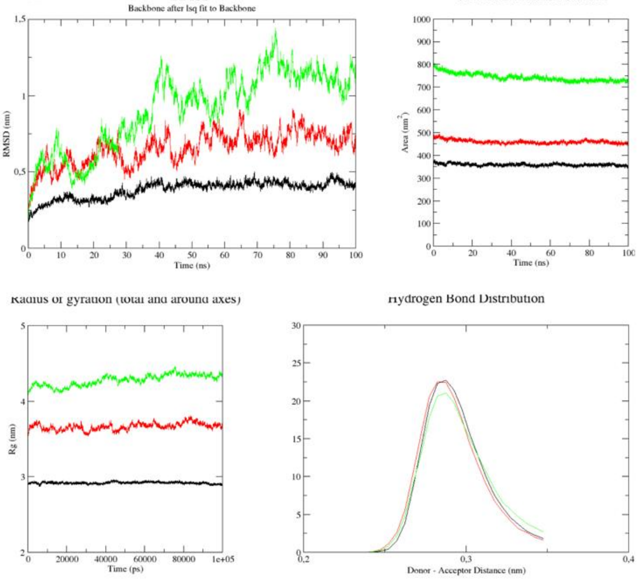
RMSD
Backbone after lsq fit to Backbone
RMSD (nm)
Time (ns)
Solvent Accessible Surface
Area (nm^2)
Time (ns)
Radius of gyration (total and around axes)
Rg (nm)
Time (ps)
Hydrogen Bond Distribution
Donor - Acceptor Distance (nm)

F

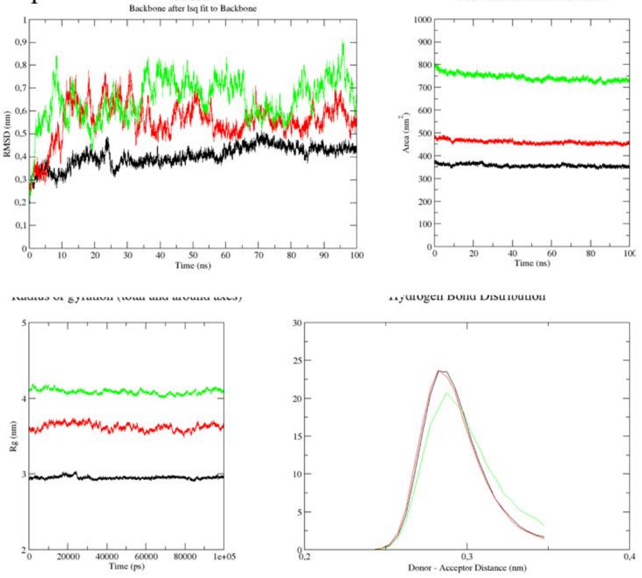
RMSD
Backbone after lsq fit to Backbone
RMSD (nm)
Time (ns)
Solvent Accessible Surface
Area (nm^2)
Time (ns)
Rg (nm)
Time (ps)
Donor - Acceptor Distance (nm)

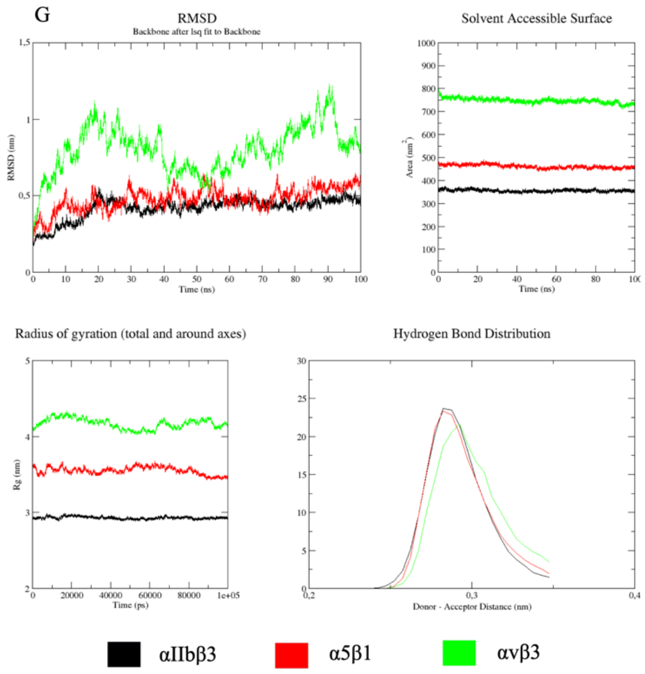

Figure S1. RMSD, SASA, Rg and H bonds values for Pep2 (A), Pep4 (B), Pep5 (C), Pep6 (D), Pep7 (E), Pep9 (F), and Pep10 (G).
